# Psychoactive substances use before suicide: detailed analysis of all cases that occurred in the Brazilian Federal District in a 10-years period

**DOI:** 10.1186/s12888-022-04082-z

**Published:** 2022-07-14

**Authors:** Juliano de Andrade Gomes, Diego Mendes de Souza, Karina Diniz Oliveira, Andrea Donatti Gallassi

**Affiliations:** 1 Forensic Institute, Civil Police of the Brazilian Federal District (PCDF), Brasilia, DF Brazil; 2 Division of Psychiatry, School of Medical Sciences, University of Campinas (Unicamp), Campinas, SP Brazil; 3 Postgraduate Program of Sciences and Health Technology, Faculty of Ceilândia (FCE), University of Brasilia (UnB), Centro Metropolitano 1, Conjunto A-Ceilândia Sul, Brasília, DF ZIP 72220-900 Brazil; 4 Center of Drugs and Associated Vulnerabilities, Faculty of Ceilândia (FCE), University of Brasilia (UnB), Brasilia, DF Brazil

**Keywords:** Psychoactive substances, Suicide, Suicidal behavior, Suicide prevention, Epidemiology, Brazil

## Abstract

**Background:**

Psychoactive substances (PASs) are an important risk factor for suicide. This study investigated the sociodemographic characteristics, data related to the suicidal behavior, the methods employed, the circumstances of the events, and the use of PASs before dying in all suicides that occurred between 2005–2014 in the Brazilian Federal District, comparing cases with positive and negative detection for PASs in the *post-mortem* analysis to identify groups at greatest risk.

**Methods:**

A population-based, observational, cross-sectional study with an analytical aspect was conducted with suicides cases collected from local police, which toxicological examination was performed (headspace gas chromatographic-mass spectrometry-HS-GC/MS) for detection of ethanol and methanol in blood samples; immunoassay for other substances (cocaine, marijuana, benzodiazepine).

**Results:**

The results showed that the increase in the suicide rate was 10 × greater than the population growth, and 44% of the individuals used PASs before suicide. Individuals are more likely to die by suicide at home, be male, have tried before, and change their behavior days before death; they choose to hang as the method and are influenced by alcohol.

**Conclusion:**

Identifying what sociodemographic characteristics are associated with a fatal suicide attempt among individuals who use PASs and those who do not use and those who have/do not have mental disorders and what methods are employed could be employed as a path to better interventions. Thus, prevention actions could be planned and directed to individuals with greater risk.

**Supplementary Information:**

The online version contains supplementary material available at 10.1186/s12888-022-04082-z.

## Background

The use of alcohol and other drugs is related to a set of consequences affecting one's health [1, 2]. A study review has demonstrated an association between the use of psychoactive substances (PASs) and mental health problems [3]; in recent decades, the literature has been shown an increased risk of suicide among individuals who consume alcoholic beverages [4] and/or use other drugs [5, 6]. PASs are one of the most critical risk factors for suicide, as it is often related to predisposing and precipitating factors of suicide attempts (SA), such as mental disorders. Reviews of cohort studies [7, 8] have shown that substance use is strongly associated with suicide. Indeed, substance use disorders and mood disorders are the most prevalent mental disorders among fatal SA, as documented by more than 20 major psychological autopsy projects [9, 10].

Considering the effects of the most prevalent PASs – depressant, stimulant, and sedative – may be associated with impulsivity, disinhibition, aggression, and sedative drugs, feelings of sadness, despair, and impaired cognition. These effects, alone or combined with the simultaneous use of PASs, may mediate suicide-related behaviors; alcohol/drug addiction is also associated with a higher risk of dying by suicide [11]. The main analytical techniques used to perform the toxicological tests to detect the use of these PASs among those who die are Gas Chromatography-Mass Spectrometry (GC–MS) and High-Performance Liquid Chromatography (HPLC) [12, 13].

The global age-standardized suicide rate was 9.0 per 100,000 population for 2019, resulting in more than 700 thousand deaths per year. More than three-quarters of these fatalities occur in low- and medium-income countries, such as Brazil. Moreover, more than 20 non-fatal SA for every fatal SA [14].

In the Brazilian Mortality Information System – Ministry of Health – there were 106,374 deaths by suicide between 2007 and 2016; in 2016, the rate reached 5.8 per 100 thousand people, with 11,433 deaths reported due to this cause [15]. The suicide rate in the Brazilian Federal District is similar to the national average, with 5.5 suicides for every 100 thousand residents in 2017 [16]. Brazilian Federal District is the most populous city in the Midwest region of Brazil and the second Brazilian region (among five) with the highest prevalence of alcohol use in adults and adolescents [17].

In addition to the association between suicide, use of PASs, and/or the occurrence of mental disorders, there is a gap in the current literature to identify which other characteristics are associated with suicidal behavior (SB) and that can lead to fatal SA. Identifying sociodemographic characteristics, methods employed, recent behavior change, possible reasons for the suicide, and when/where it happened becomes essential to recognize groups at greater risk and plan directed actions [18–20]. In Brazil, it has been a consistent overall prevalence of suicide by males, with the most common suicides methods: hanging, injury by firearms, and substance intoxication in the city of São Paulo from 2011 to 2015 [21], as well in the Los Angeles County, in the US, where hangings were a frequent cause of death by suicide from 2016 – 2020 and commonly occur at home [22]. A critical review of studies has also shown that owning a gun might potentially impact overall suicide rates due to its ease of use and lethality [23]. Approximately 90% of those who attempt suicide and survive do not later die by suicide; however, attempts with a gun are usually fatal [24].

Suicidal ideation (SI) can be highly complex and is usually challenging to comprehend among those who died by suicide such motivation, unless there is a suicide note or any relic found at the crime scene. However, there is evidence that leaving a note among those who died by suicide does not represent all cases, and it is associated with specific characteristics and methods [25]. An alternative method is to perform a psychological autopsy with family members and close acquaintances to understand possible changes in recent behavior and possible reasons for a fatal SA [26].

Therefore, this study aimed to describe the sociodemographic characteristics, data related to the suicidal behavior (SA, recent behavior change, reasons for the suicide), methods employed, circumstances of the events (place, day of week and of month, day period), and the use of PASs before dying, in all suicides that occurred in a ten-years period (2005 – 2014) in the Brazilian Federal District, comparing cases with positive and negative detection for PASs in the *post-mortem* toxicological analysis, to identify groups at greatest risk.

## Methods

A population-based, observational, cross-sectional study with an analytical aspect (forensics analysis) was performed. In the Brazilian Federal District, all people who died between January 1^st^, 2005, and December 31^st^, 2014, whose primary cause of final death was suicide, were included. Data were collected at the Civil Police of the Brazilian Federal District (PCDF) from (*i*) Police Station reports, accomplished by investigators during the interview with relatives and/or witnesses, and (*ii*) Coroners reports at the Legal Medicine Institute produced during the performance of forensics analysis on the suicide victim's body (toxicological examination). A trained team evaluated all records of the Legal Medicine Institute and the police reports to understand the characteristics related to suicide. Then, two independent appraisers filled out a standardized form with the information provided in the documents.

In Brazilian Federal District, all deaths by suicide should undergo toxicological examination. In some cases, it does not occur due to technical problems and/or because the victim was sent to the hospital and received medical treatment, dying sometime later, rendering the examination impossible to be accomplished at the time of death by suicide. Thereby, only cases with toxicological tests performed for alcohol and other drugs were selected for the present study. Toxicological tests were performed by the team of the Medical-Legal Institute of PCDF. Headspace gas chromatographic-mass spectrometry (HS-GC/MS) was used to detect ethanol and methanol in blood samples. Other substances (cocaine, marijuana, benzodiazepines, etc.) were investigated using immunoassays.

The following variables were analyzed: *day of the month* (1–10 or 11–20 or 21–31); *day of the week* (weekday or weekend); *season* (spring or summer or autumn or winter); *period* (day '6 h-18 h' or night' 18 h-5h59h’); *place* (own residence or others place); *age group* (adolescent '12–17 years old' or young adult '18–29 years old' or adult '30–59 years old’ or elderly ‘ ≥ 60 years old’); *sex* (male or female); *level of education* (illiterate or elementary School or high school or higher education); *marital status* (married ‘any relationship in which the person lives together maritally’ or not married ‘any relationship in which the person does not live with another maritally’); *previous SA*? (yes or no); *recent behavior change?* (yes or no); *what has changed?* (became aggressive or became depressed or became unstable); *skin color* (white or black/ ‘pardo’); *Body Mass Index – BMI* (underweight ‘ < 18.5 kg/m^2^’ or ideal weight range’18.5–24.9 kg/m^2^’ or overweight’25–29.9 kg/m^2^’ or obesity ‘ ≥ 30 kg/m^2^’); *profession* (unemployed or public servants or construction or others); *reasons of the suicide* (drug addiction or crime of passion or mental disorder or others); *method of the suicide* (hanging or firearm or poison or jumping from eight or others); *drug use before to die?* (yes or no); the *number of drugs used* (1 or ≥ 2); *type of drugs* (cocaine or benzodiazepine or marijuana or others); *alcohol use?* (yes or no) and *Blood Alcohol Concentration – BAC level* (0.01–0.49 g/l or 0.50–0.99 g/l or 1.00–1.49 g/l or 1.50–2.99 g/l or 3.00–3.99 g/l or ≥ 4.00 g/l). Data related to the act of suicide (previous SA?; recent behavior change?; what has changed?; and reasons for suicide) were collected from police staff who interviewed the victims' relatives. Drug addiction (one of the three options of the variable ‘reasons of the suicide’) was defined according to the report of the victims' relatives to the police staff (‘he/she had drug problems’; ‘he/she was an alcoholic/addicted’), and the variables' type of drug' and 'alcohol use' were the substances detected in the *post-mortem* toxicological analysis (Table S1, in Supplementary Material).

The difference between the annual increase in the suicide rate (per 100 thousand residents) of the individuals who used PASs and the population growth of the Brazilian Federal District was evaluated using generalized linear mixed models [PROC GLIMMIX procedure in the Statistical Analysis System (SAS) software]. Descriptive analysis [absolute and relative (percentage) frequency of each variable] was performed using the SAS software. Frequency values were compared to those known for the Brazilian Federal District (test percent), and *p*-values were determined using the χ^2^ test for specified proportions (a *p*-value < 0.05 was considered statistically significant). Relative risk (RR) was determined by dividing the percentage obtained for each variable by the known percentage for the Brazilian Federal District. When a value was unknown for the Brazilian Federal District, the risk was determined by dividing the percentage of the category analyzed by the reference category.

To perform Pearson's correlation for each pair of variables (p < 0.05 to be considered significant) using the OriginPro 9 software, all qualitative variables were converted into ordinal variables, and the data were then normalized (means were used for unknown values). Odds ratios were calculated for the population studied, with individuals who died by suicide and did not use PASs before dying considered the control group.

Principal component analysis (PCA) is an exploratory data analysis tool that reduces the dimensionality of the data while maintaining most of the initial variance. In the present study, PCA was performed in OriginPro 9 to obtain a synthetic, global view of the data and determine (based on loadings) what variables were more or less correlated, as well as identify those that contributed most to the first main components, which are the most informative. Five components were considered, as performed in a previous study [27]. PCA was also used to evaluate trends in the variables assessed to characterize particular groups of individuals who died by suicide. The correlation matrix was used to complement the trends found in the PCA.

## Results

A total of 1,088 suicides occurred in the Brazilian Federal District between 2005 and 2014. In 308 cases, the toxicological examination was not performed, therefore excluded from the present study. Thus, 780 cases comprised the sample (344 with positive results for PASs use before dying and 436 with negative results), as shown in Diagram S1 (Supplementary Material).

**Fig. 1:**
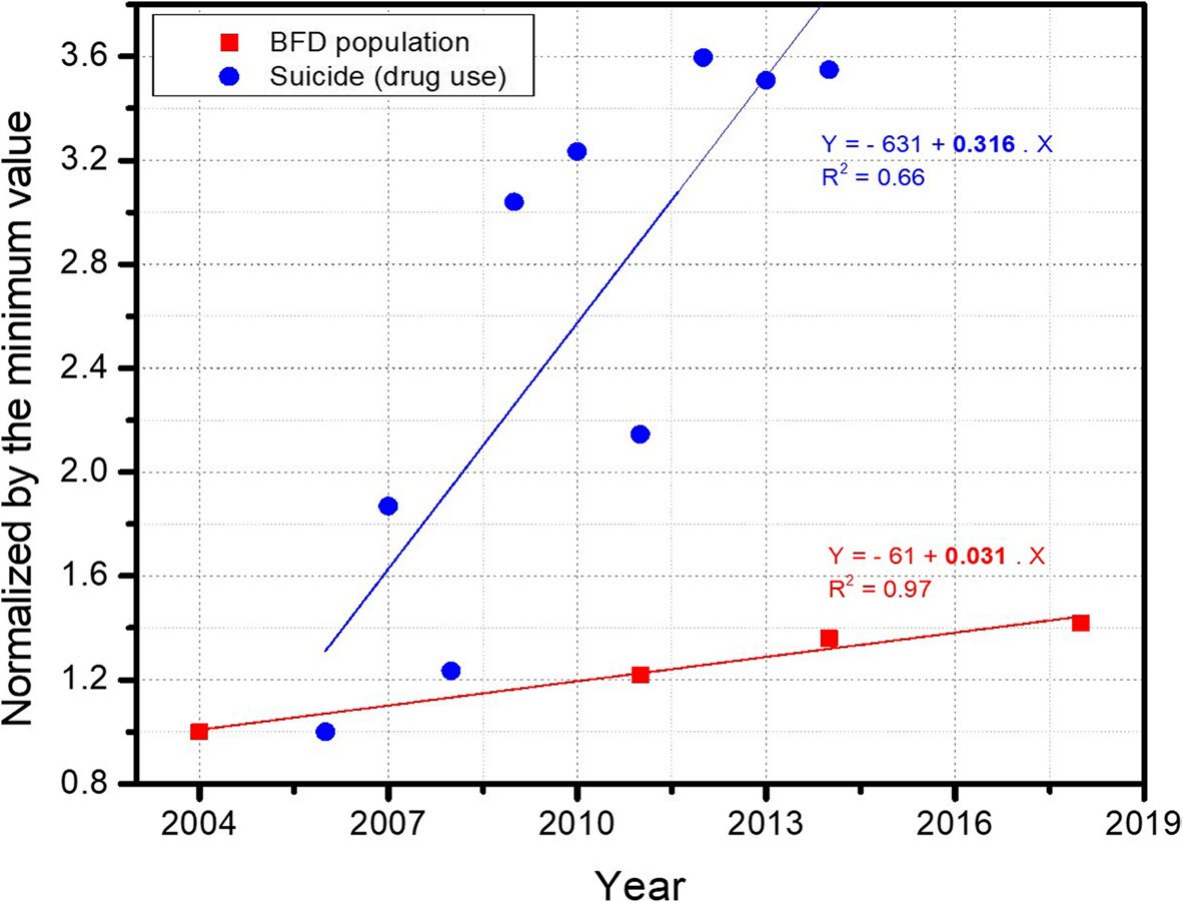
Brazilian Federal District population (red square symbols; 2004 – 2018) and suicide rate of individuals who used PASs (psychoactive substances; blue circles; 2005 – 2014) normalized by the minimum value of each category as a function of year. Lines represent the best linear fit

Figure 1 shows the Brazilian Federal District population and suicide rate of those who used PASs normalized by the minimum value of each category as a function of the year and the best linear fits and respective equations. The suicide rate of those who used PASs was 10 times higher than the population growth rate (p = 0.0087, in slope comparison using GLIMMIX procedure).

The descriptive analysis of the data (Table 1) showed that the difference in the parameters analyzed within each of these variables – *Day of the week*, *Period*, *Place*, *Age group*, *Sex*, *Level of education*, *Previous* *SA?*, *Recent behavior change?*, *What has changed?*, *Skin color* and *BMI* – were significantly relevant (p < 0.05, χ^2^ test for specified proportions). On the other hand, *Day of the month*, *Season*, and *Marital status* were non-significant.

**Table 1:** Descriptive analysis, χ.^2^ test for specified proportions, and relative risk (RR) for some variables considered in this study. Brazilian Federal District, 2005 – 2014

	**Parameter**	**Frequency**	**Percent**	**Test percent**	p*	**RR**
**Day of month** (*n* = 344)	1—10	121	35.17	32.88	0.545	1.07
	11—20	114	33.14	32.88		1.01
	21—31	109	31.69	34.24		0.93
**Day of week** (*n* = 344)	Weekday	207	60.17	71.43	** < 0.0001**	0.84
	Weekend	137	39.83	28.57		1.39
**Season** (*n* = 344)	Spring	101	29.36	25.00	0.264	1.17
	Summer	76	22.09	25.00		0.88
	Autumn	85	24.71	25.00		0.99
	Winter	82	23.84	25.00		0.95
**Period** (*n* = 344)	Day	150	43.60	50.00	**0.018**	0.87
	Night	194	56.40	50.00		1.13
**Place** (*n* = 325)	Own residence	240	73.85	50.00	** < 0.0001**	1.48
	Others place	85	26.15	50.00		0.52
**Age group** (*n* = 344)	Adolescent	16	4.65	12.57	** < 0.0001**	0.37
	Young adult	121	35.17	28.86		1.22
	Adult	191	55.52	49.14		1.13
	Elderly	16	4.65	9.44		0.49
**Sex** (*n* = 344)	Male	288	83.72	47.87	** < 0.0001**	1.75
	Female	56	16.28	52.13		0.31
**Level of education** (*n* = 125)	Illiterate	1	0.80	11.91	** < 0.0001**	0.07
	Elementary School	42	33.60	33.03		1.02
	High school	53	42.40	28.68		1.48
	Higher education	29	23.20	26.37		0.88
**Marital status** (*n* = 227)	Married	95	41.85	44.87	0.360	0.93
	Not married	132	58.15	55.13		1.05
**Previous suicide attempt?** (*n* = 74)	Yes	69	93.24	50.00	** < 0.0001**	1.86
	No	5	6.76	50.00		0.14
**Recent behavior change?** (*n* = 110)	Yes	97	88.18	50.00	** < 0.0001**	1.76
	No	13	11.82	50.00		0.24
**What has changed?** (*n* = 98)	Became aggressive	32	32.65	33.33	** < 0.0001**	0.98
	Became depressed	51	52.04	33.33		1.56
	Became unstable	15	15.31	33.33		0.46
**Skin color** (*n* = 272)	White	49	18.01	41.86	** < 0.0001**	0.43
	Black/*pardo*	223	81.99	58.14		1.41
**BMI**^a^ (*n* = 301)	Underweight	19	6.31	25.00	** < 0.0001**	0.25
	Ideal weight range	179	59.47	25.00		2.38
	Overweight	79	26.25	25.00		1.05
	Obesity	24	7.97	25.00		0.32

^*^ *p*-value for χ^2^ test for specified proportions; RR: relative risk; *p*-values in bold are statistically significant (*p* < 0.05)

^a^ Not possible to obtain a specific proportion for BMI (Body Massa Index) data of the BFD population; thus, equal distribution was used among parameters

Analyzing the relative risk (last column in Table 1), we can establish the probability of suicide within each investigated variable, dividing the value of one parameter by another. For example, the probability of a person using PASs and dying by suicide on the weekend was 1.6-fold greater than those who die by suicide on the weekday; at home was 2.8-fold greater than in any other place, and being a man was 5.6-fold greater than being a woman.

Table 2 shows the descriptive analysis for other variables. Considering employment, the most vulnerable groups were tradespeople and liberal professionals (29.89%), and unemployed individuals (28.80%). The two primary reasons for the suicide were drug addiction (34.75%) and crime of passion (28.75%). It is noteworthy that family members may have been informed of more than one reason, and all of them were considered; therefore, the number of times the term appeared was counted. Hanging was the method of suicide chosen by more than half of the individuals (62.79%), followed by using a firearm (17.44%). Among those who used a firearm, the head region was the most common anatomical site chosen (86.15%).

**Table 2:** Descriptive analysis and risk for some of the variables considered in this study. Brazilian Federal District, 2005 – 2014

	**Parameter**	**Percent**	**Risk**
**Profession** (*n* = 184)	Tradespeople and liberal professionals	29.89	2.20
	Unemployed	28.80	2.12
	Public servants	15.22	1.12
	Construction	12.50	0.92
	Others	13.59	1.0 ^r^
**Reasons for the suicide** (*n* = 264)	Drug addiction	34.75	2.28
	Crime of passion	28.75	1.89
	Mental disorder	21.25	1.39
	Others	15.25	1.0 ^r^
**Method of the suicide** (*n* = 344)	Hanging	62.79	19.62
	Firearm^a^	17.44	5.45
	Poison	8.43	2.63
	Jumping from height	8.14	2.54
	Others	3.20	1.0 ^r^
**Drug use?** (*n* = 315)	Yes	56.19	1.28
	No	43.81	1.0 ^r^
**Number of drugs used** (*n* = 177)	1	83.05	4.90
	≥ 2	16.95	1.0 ^r^
**Types of drugs** (*n* = 208)	Cocaine	56.25	12.99
	Benzodiazepine	20.19	4.66
	Marijuana	19.23	4.44
	Others	4.33	1.0 ^r^
**Alcohol use?** (*n* = 339)	Yes	71.68	2.53
	No	28.32	1.0 ^r^
**BAC level (g/l)** (*n* = 339)	0.01—0.49	14.40	6.99
	0.50—0.99	21.40	10.39
	1.00—1.49	20.58	9.99
	1.50—2.99	34.98	16.98
	3.00—3.99	6.58	3.19
	≥ 4.00	2.06	1.0 ^r^
**Association between alcohol and other drug use**^b^		22.09	-

The risk was obtained by dividing the percentage of category analyzed by reference category ^r^

*BAC* Blood alcohol concentration

^a^ 86.15% of suicides preferred to shoot in the head region and another 13.85% in the thoracoabdominal area

^b^ The percentage that used both before diying by suicide

In the sample studied, 71.68% used alcohol, 56.19% used other drugs, and 22.09% used both. Most individuals who used PASs (except alcohol) used only one type of them (83.05%), and the most common was cocaine (56.25%). Among those who used alcohol before dying by suicide, the mean dose was 1.49 g of alcohol per liter of blood, whereas nearly 35% had a blood alcohol concentration between 1.5 g/l and 2.99 g/l.

Table 3 shows Pearson’s correlation coefficients for the linear correlation between pairs of variables. The most relevant correlations are presented below and discussed in the next section. Younger people were more likely to die by suicide on the weekend, whereas older people were more likely on a weekday. Over time, individuals died by suicide more during the day, average age increased (see inset of Table 3), and more individuals had recently demonstrated a behavior change. Married people were more likely to die by suicide at night, whereas unmarried people during the day. With the increase in age, people who died by suicide were married, had white skin color, had high blood alcohol concentration, and did not use other drugs. While men chose to hang as the primary method of suicide, women preferred jumping from a height or poison. Individuals who died by suicide in their own homes preferred the hanging method. Individuals with previous SA were less likely to use PASs before dying by suicide. Those who recently changed their behavior before dying by suicide were more likely to have a higher blood alcohol concentration. Individuals who jumped from a height were more likely to be under the influence of a PAS (except alcohol).

**Table 3:**
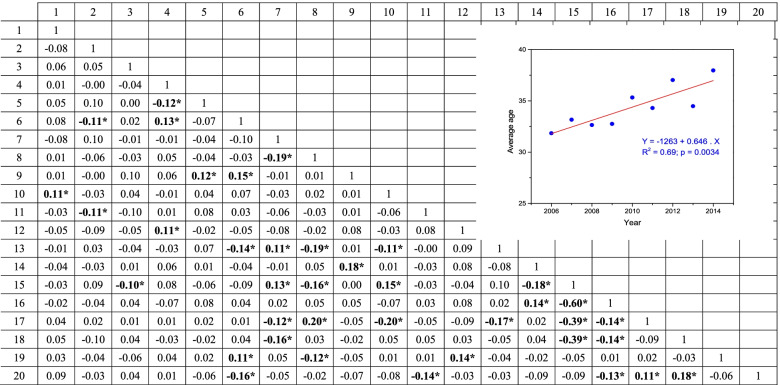
Pearson’s correlation coefficients for linear correlation between pairs of variables. Inset shows an increase in average age throughout the study period. Brazilian FederalDistrict, 2005 – 2014

2-tailed test of significance used; acorrelation significant at 0.05 level

1. Day of the month; 2. Day of the week; 3. Month; 4. Year; 5. Period; 6. Age; 7. Sex; 8. Level of education; 9. Marital status; 10. Suicide in own home; 11. Previous attempt?; 12. Behavior changed recently?; 13. Skin color; 14. Body Mass Index; 15. Hanging; 16. Firearm; 17. Jumping from height; 18. Poison; 19. Blood Alcohol Content (BAC); 20. Drug use

The odds ratios show the risk of dying by suicide among individuals with and without PASs according to the variables shown in Table 4. Statistically significant differences were found for seven of the 17 variables studied. Individuals who used PASs before dying by suicide were more likely to have a fatal SA on weekends. Individuals who did not use PASs (except alcohol) were more likely to die by suicide during the day, whereas those who used PASs were more likely to die by suicide at night. Among the age groups, the elderly and adolescents were more likely not to have used PASs (except alcohol), and women were more likely not to have used any PASs before dying by suicide. Among occupations, construction workers were more likely to use PASs before committing suicide. Individuals whose reason for committing suicide was drug addiction were more likely to use drugs before committing suicide. Individuals with a mental disorder were less likely to use PASs (except alcohol). Individuals who chose poison as the suicide method were less likely to be under the influence of PASs before dying by suicide.

**Table 4:** Odds ratios for variables of interest comparing individuals who used PASs to those who did not. Brazilian Federal District, 2005 – 2014

	**Parameter**	**Frequency / Total**		**Odds ratio**	**95% CI**	***p***
		**Sample**	**Control**			
**Day of month**	01—10	121/344	145/436	1.06	0.80—1.40	0.6944
**Day of week**	Weekend	137/344	110/436	1.58	1.18—2.11	**0.0019**
**Season**	Summer	76/344	102/436	0.94	0.68—1.31	0.7330
	Winter	82/344	112/436	0.93	0.68—1.28	0.6449
**Period**	Day	150/344	287/436	0.66	0.52—0.84	**0.0009**
	Night	194/344	149/436	1.65	1.28—2.13	**0.0001**
**Place**	At home	240/325	304/415	1.01	0.81—1.26	0.9435
**Age group**	Adolescent	16/344	40/436	0.51	0.28 – 0.92	**0.0257**
	Elderly	16/344	63/436	0.32	0.18 – 0.57	**0.0001**
**Sex**	Female	56/344	106/436	0.67	0.47—0.95	**0.0261**
**Level of education**	Elementary School	42/125	68/161	0.80	0.51—1.25	0.3192
	High school	53/125	46/161	1.48	0.94—2.35	0.0918
**Marital status**	Married	95/227	123/275	0.94	0.68—1.29	0.6841
**Previous suicide attempt?**	Yes	69/74	82/88	1.00	0.64—1.56	0.9977
**Recent behavior change?**	Yes	97/110	122/137	0.99	0.69—1.43	0.9581
**What has changed?**	Became aggressive	32/98	30/123	1.34	0.76—2.35	0.3110
	Became depressed	51/98	74/123	0.86	0.55—1.35	0.5226
**Skin color**	White	49/272	92/372	0.73	0.50—1.07	0.1024
**BMI**	Overweight	103/301	146/362	0.85	0.63—1.14	0.2748
**Profession**	Construction	23/184	12/231	2.41	1.17—4.96	**0.0175**
	Unemployed	53/184	56/231	1.19	0.78—1.81	0.4239
**Reasons for the suicide**	Crime of passion	115/400	113/483	1.23	0.92—1.64	0.1658
	Drug addiction	139/400	116/483	1.45	1.09—1.91	**0.0097**
	Mental disorder	85/400	151/483	0.68	0.51—0.91	**0.0108**
**Method of the suicide**	Hanging	216/344	240/436	1.14	0.90—1.44	0.2658
	Firearm	60/344	66/436	1.15	0.79—1.68	0.4615
	Poison	29/344	60/436	0.61	0.38—0.98	**0.0390**
	Jumping from height	28/344	51/436	0.70	0.43—1.13	0.1405

*CI* confidence interval

*p*-values in bold statistically significant (*p* < 0.05)

The PCA yielded five uncorrelated components (Table S2, in Supplementary Material), enabling a global, synthetic data view. These components had eigenvalues of 1.61, 1.43, 1.29, 1.28, and 1.17 and explained 9.47%, 8.40%, 7.60%, 7.56%, and 6.90% of the total variance in the initial data, respectively. The components were characterized as follows:

- Component 1 – suicide method: men, young people, individuals with a lower education level, and individuals with black/*pardo* skin color chose hanging; women, older people, individuals with a higher education level, and white-skinned individuals chose other suicide methods;
- Component 2 – drug group: individuals with higher levels of schooling and younger people preferred using PASs (except alcohol); older people and those with lower levels of education chose alcohol;
- Component 3 – suicidal thinking (characteristics of individuals who have non-fatal SA); preferred suicide at night, away from home, and at the beginning of the month; low loadings for *alcohol use* and other *drug use* and opposite directions to *the previous attempt* suggest that recidivism and suicide do not depend on drug use, which confirms the belief in suicide;
- Component 4 – behavioral traits: individuals who had a recent behavioral change due to delusion, depression, etc., tended to commit suicide outside the home, during the day, and in the first months of the year; loading associated with *recent behavior change* was in the opposite direction of marital status, which means that the unexpected change in behavior predominantly occurred among single people;
- Component 5 – premeditation: over the years, suicide occurred more during the day and at the beginning of the month, independent of the dosage of PASs used (except alcohol); these tendencies indicate a higher degree of forethought, which may explain the higher increase in suicides compared to the rise in the population.

Figure S1 (Three-dimensional PCA graph considering the first three principal components, in Supplementary Material) presents the three-dimensional PCA graph considering the first three main components, reinforcing the discussion established in the previous paragraph (individual component). Hanging (Hang.), sex (Gen.), and skin color (Skin) are vectors with approximately the same dimension positioned in the right hemisphere of the figure and are therefore correlated. Moreover, these vectors are in the opposite direction (negatively correlated) to the level of education (Edu) and age (Age), justifying the discussion related to Component 1 (suicide method). Drug use (Drug), level of education (Edu), and age (Age) are on the left side of the graph, validating Component 2 (drug group). Previous attempts (Prev. Att.?) and period (Period) are directed to the central lower part of the graph and are positively correlated. Moreover, these vectors are opposite to place (Resid.) and day of the month (day-m), corroborating what has been discussed for suicidal thinking (Component 3).

**Fig. 2:**
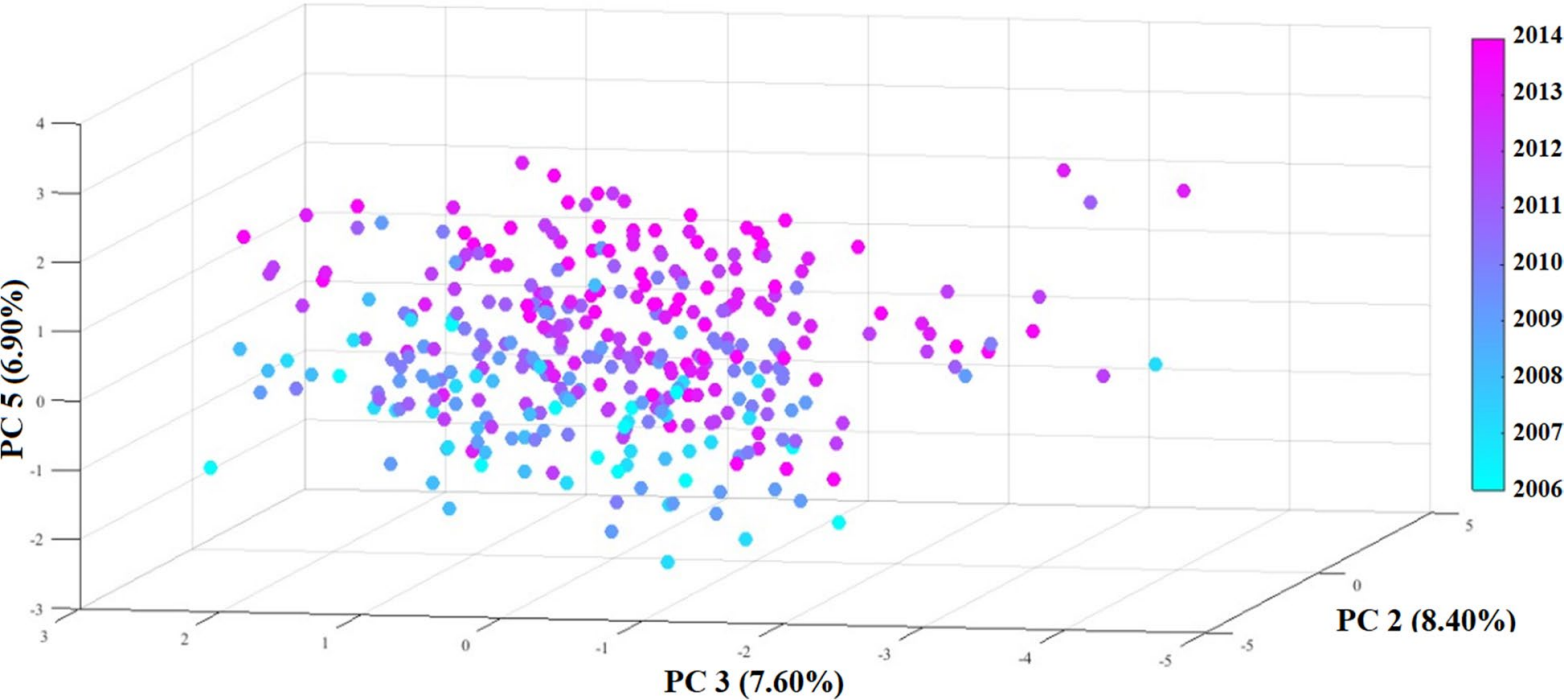
4D graph of scores of 344 individuals on the first three components (PC1 x PC2 x PC3) in relation to year, represented on color scale. Brazilian Federal District, 2005 – 2014

Figure 2 presents a 4D PCA graph. The three axes correspond to the principal components with a higher coefficient for the variable year, and a fourth dimension (color) corresponds to the year.

The graph of the scores shows that the data are very dispersed due to randomness and the peculiarities of each suicide event. However, using the degradation, although considerable dispersion was maintained over the historical series, the spatial position of the data changed, with lower values, on average, for Component 3 and higher values for Components 2 and 5. This indicates that the suicide characteristics among PASs users changed over time, albeit slowly. Correlating this position change of the dispersion of the points with the factors attributed to the components, one may infer that the increase in suicide associated with the alcohol use (PC2) was accompanied by increases in the forethought of suicide (PC5) and the number of individuals who had previously SA (PC3). There is no way to establish a relationship among these three trends since, algebraically, the components are orthogonal (independent of one another). Therefore, this change in characteristics may be related to different groups that shaped this change in the individuals who died by suicide and had used PASs before. Furthermore, the low accumulated variance in the first five components (39.93%) supports the notion that suicide is a dependent, multivariate event that the analysis of a few effects cannot fully explain.

## Discussion

The present study results show that PASs was an important element in the cases of suicide that occurred in the Brazilian Federal District between 2005 and 2014, confirming the established association between PASs use and suicide reported in previous studies [28, 29]. This association may be explained by the psychoactive effects of certain substances, which can trigger potentially lethal impulsive behavior. As shown in Fig. 1, the suicide rate of people who have used PASs before to die by suicide is growing at approximately 10 × the population growth, making this event alarming. Therefore, studies like this are essential to establish standards to reduce such cases.

Alcohol is a substance with psychoactive effects that can increase impulsivity and accentuate SB. A recently published study conducted in Brazil showed that being male and having cocaine identified in the toxicological test were the factors that showed a strong association with abusive alcohol beverage consumption among those who died by suicide [30]. According to the present results, the mean dose used before dying by suicide was 1.49 g of alcohol per liter of blood, and the blood alcohol concentration was between 1.5 g/l and 2.99 g/l in nearly 35%. According to Larini [31], this dose can cause disorientation, mental confusion, dizziness, an exaggerated emotional state (fear, annoyance, or distress), sensation/perception disturbance, balance deficit, muscle incoordination, and difficulty speaking. Another study concluded that, due to the acute effect of alcohol on neurotransmitters and cognitive functions, the use of this substance could drive SA through different mechanisms, enhancing dysphoria, aggressiveness, impulsivity, and disinhibition [32]. A vicious circle can be created between PASs/impulsivity/aggression/SB and the fact that it is often unclear whether the individual has used a substance to facilitate the transition to the act or whether the fact of chronically consuming a substance – drug addiction – predisposes, for a variety of reasons, to SB [33]. Indeed, there is a dose–response relationship between the amount of alcohol consumed and the risk of suicide [29]. These mechanisms could explain the amplifying effect of alcohol use on suicide mortality; when a stressful event occurs, high alcohol intake affects the central nervous system, which may amplify negative emotions [32].

The prevalence of suicide was higher among men than women, and men with a skin color black/*pardo* were 3.3-fold more likely to commit suicide. Data from the Brazilian Health Ministry show that the death rate due to suicide was 6.13 per 100 thousand people in 2016 (9.8 for men and 2.5 for women); moreover, men and black/*pardo* skin color were the most numerous groups among deaths by suicide in 2018 [15]. In Brazil, black and *pardo* individuals constitute a more socially vulnerable group, which may be a risk factor for suicide. An Australian survey that associated individual characteristics with lethal methods of suicide found that individuals with a more precarious social network and greater social vulnerability were at greater risk of dying by suicide [18]. Another study found that experiencing gendered racism significantly increased the risk for SI or SB among women [19].

The greater proportion of men among cases of suicide has also been reported in other studies; some of them show that, although women are more likely to SA more often and perform acts of self-mutilation, men tend to use more violent methods with greater lethality [34]. The use of medications and poisoning as a method of suicide is more socially acceptable for women than men, as confirmed in the present study, in which poisoning was the method most used by women. Other factors may influence the lower mortality rate due to suicide among women. Women generally have more protective factors, such as stronger religious beliefs, a solid and more effective social network, and a greater willingness to seek help for their mental disorders and SI [35].

Clinical and sociodemographic characteristics may distinguish the choice of method. A study analyzed 8,103 deaths by suicide between 2000 and 2013 and found that hanging accounted for 48.4% of the cases; moreover, males, indigenous people, and individuals who resided in rural and remote areas were more likely to die by hanging [18]. Given the easy availability of hanging materials and the difficult access to firearms among younger age groups, individuals who feel angry due to an interpersonal conflict in combination with alcohol use may be impulsively driven to hang themselves, as other methods would require a more extended preparation [36]. The results of the present study identified a similar profile, as men, young people, individuals with a lower education level, and individuals with black/*pardo* skin color chose the hanging method.

A previous Brazilian study involving an epidemiological analysis of suicide indices registered in the country between 1980 and 2006 found that the main sociodemographic characteristics of the individuals who die by suicide were a low educational level and being single. Also, the survey showed that one's own home was the primary location for suicide (51%), and among the deaths that occurred in the home, 64.5% were by hanging, and 17.8% were by firearms [37].

Individuals who jumped from a height to die by suicide were usually under the influence of PASs (except alcohol) in the present study. The use of PASs, especially cocaine, can increase the lethality of SB; individuals under the effect of cocaine/crack tend to SA using more lethal methods than those that have not used [38]. An American study analyzing suicides by hanging and firearms to explore how specific drugs are associated with the method of suicide as a function of demographic and social characteristics found that the association between the use of cocaine/opiates and a violent method varied with the level of schooling [28]. In our data, individuals with higher levels of education and younger people opted to use PASs (except alcohol), whereas older people and those with lower levels of schooling preferred alcohol.

In the sample, the use of PASs was non-significant among suicides by less violent methods, such as poisoning, and individuals who had recently changed their behavior preferred not to use drugs. Studies have shown that mental disorders constitute an important risk factor for SB, independently of the PASs use. A fatal and a non-fatal SA are more frequent among patients who suffer mood disorders than the other psychiatric disorders in comparison who do not present any mental health problems [39]. Anxiety and obsessive–compulsive disorder may also be associated with the attempted suicide of high lethality, mainly when associated with the use of PASs [40].

The present findings suggest that previous SA is a significant risk factor for dying by suicide. According to the World Health Organization, the main risk factors are suffering from a mental disorder and having a history of non-fatal SA [41]. Individuals with prior attempts have a 40-to-66-fold greater risk of dying by suicide than the general population [42].

The multivariate analysis of the data using PCA [43, 44] indicated a gradual change in the suicide profile, pointing to an increasing contribution of new determinant factors for suicide, forethought for suicide, and prior attempts, which underscores the need for the adoption of preventive public health policies explicitly directed at this population.

Suicide is a mental health problem that continues to pose a challenge for the scientific community and healthcare providers with regard to the identification of risks that can assist in the implementation of prevention measures. The PASs use and mental disorders are strongly associated with a fatal SA. However, these data per se are not capable to prevent the occurrence of so many deaths every year.

A comprehensive analysis of sociodemographic characteristics and suicide methods would better predict future risk groups and plan prevention measures “custom-made”. Identifying what sociodemographic characteristics are associated with a fatal SA among individuals who use PASs and those who do not use them, and those who have/do not have mental disorders and what methods are employed could be a path to better interventions. Professionals who work in specialized mental health/drug addiction services, as well as those in the primary care, can identify SB in large samples and among community-dwellers using easy-to-apply screening instruments, such as the PHQ-9 scale for initial screening of the presence of depression [45], the Beck SSI scale for the evaluation of the presence/intensity of suicidal ideation [46], and the Columbia Scale for the assessment of suicidal risk [47]. These prevention actions could be planned and directed to individuals with greater risk.

### Limitations and strengths

Regarding limitations, we could not fit all variables to all individuals since the data were collected from police and coroners' reports, which did not always have all the fields correctly filled out. Data such as *previous attempt*s, *recent behavior change*s, and *reason*s for the suicide were reported by relatives at the police station. While the coroner provided the skin color of the individuals investigated in the present study, the skin color of the population of the Brazilian Federal District is self-declared. It was not possible to obtain the BMI for the Brazilian Federal District population to compare it with the χ^2^ test for specified proportions.

The article's strengths are the sample size, the considered period, the post-mortem analysis with the toxicological test performed, and the diversity of analyzed and correlated variables, making it possible to identify specific clusters of populations at higher risk to create more targeted prevention interventions.

## Conclusions

This study sought to characterize in detail the factors and characteristics associated with those who died by suicide, contributing to the better planning of preventive actions to avoid suicide deaths and care for those exposed to greater risks. However, this is a complex issue, and future studies should seek a better understanding of the factors that exert an influence on a fatal SA to improve health interventions.

## Supplementary Information

**Additional file 1:** **Diagram S1.** Cases of suicide with toxicological examination were included in the study. **Table S1.** Parameters adopted for the variables studied. Brazilian Federal District, 2005 – 2014. **Table S2.** Results of principal component analysis showing factor loadings (correlation coefficients) and variance. Brazilian Federal District, 2005 – 2014. **Figure S1.** Three-dimensional PCA graph considering the first three principal components. Brazilian Federal District, 2005 – 2014.

## Data Availability

The datasets used and/or analyzed during the current study are available at the link: http://alcooledrogas.unb.br/nossas-publicacoes

## Abbreviations

PASs: Psychoactive substances
SA: Suicidal attempt
SB: Suicidal behavior
SI: Suicidal ideation
PCDF: Civil Police of the Brazilian Federal District
HS-GC/MS: Headspace gas chromatographic-mass spectrometry
BMI: Body Mass Index
PCA: Principal component analysis

## References

[1] WHO - World Health Organization. Global status report on alcohol and health 2018. Geneva; 2018. https://www.who.int/substance_abuse/publications/global_alcohol_report/gsr_2018/en/.

[2] Lewer D, Freer J, King E, Larney S, Degenhardt L, Tweed EJ, Hope VD, Harris M, Millar T, Hayward A, Ciccarone D, Morley KI (2019). Frequency of health-care utilization by adults who use illicit drugs: a systematic review and meta-analysis. Addiction.

[3] Jane-Llopis E, Matytsina I (2009). Mental health and alcohol, drugs and tobacco: a review of the comorbidity between mental disorders and the use of alcohol, tobacco and illicit drugs. Drug Alcohol Rev.

[4] Amiti S, Behnezhad S (2020). Alcohol use and risk of suicide: a systematic review and Meta-analysis. J Addict Dis.

[5] López-Goñi JJ, Fernández-Montalvo J, Arteaga A, Haro B (2018). Suicidal ideation and attempts in patients who seek treatment for substance use disorder. Psychiatry Res.

[6] Pillon SC, Vedana KGG, Teixeira JA, Santos LA, Souza RM, Dieh A, Rassool GH, Miasso AI (2019). Depressive symptoms and factors associated with depression and suicidal behavior in substances user in treatment: focus on suicidal behavior and psychological problems. Arch Psychiatr Nurs.

[7] Harris EC, Barraclough B (1997). Suicide as an outcome for mental disorders: a meta-analysis. Br J Psychiatry.

[8] Wilcox HC, Conner KR, Caine ED (2004). Association of alcohol and drug use disorders and completed suicide: an empirical review of cohort studies. Drug Alcohol Depend.

[9] Isometsä ET (2001). Psychological autopsy studies - a review. Eur Psychiatry.

[10] Schneider B (2009). Substance use disorders and risk for completed suicide. Arch Suicide Res.

[11] Orpana H, Giesbrecht N, Hajee A, Kaplan MS (2021). Alcohol and other drugs in suicide in Canada: opportunities to support prevention through enhanced monitoring. Inj Prev.

[12] Ferrari Júnior E, Santos JBA, Caldas ED (2021). Drugs, pesticides and metabolites in forensic post-mortem blood samples. Med Sci Law.

[13] Ferrari Júnior E, Caldas ED (2022). Determination of new psychoactive substances and other drugs in post-mortem blood and urine by UHPLC–MS/MS: method validation and analysis of forensic samples. Forensic Toxicol.

[14] WHO - World Health Organization. Suicide worldwide in 2019: global health estimates. Geneva; 2021. https://www.who.int/publications/i/item/9789240026643.

[15] Brazil. Ministério da Saúde, Datasus, Estatísticas vitais – Mortalidade – 1996 a 2018, pela CID-10; 2018. http://www2.datasus.gov.br/DATASUS/index.php?area=0205.

[16] IPEA - Instituto de Pesquisa Econômica Aplicada. Atlas da violência: suicídio; 2020. https://www.ipea.gov.br/atlasviolencia/filtros-series/16/suicidios.

[17] Bastos FIPM (2017). III levantamento nacional sobre o uso de drogas pela população brasileira.

[18] Kõlves K, McDonough M, Crompton D, De Leo D (2018). Choice of a suicide method: trends and characteristics. Psychiatry Res.

[19] Perry BL, Stevens-Watkins D, Oser CB (2013). The moderating effects of skin color and ethnic identity affirmation on suicide risk among low-SES African American women. Race Soc Probl.

[20] Breet E, Goldstone D, Bantjes J (2018). Substance use and suicidal ideation and behaviour in low- and middle-income countries: a systematic review. BMC Public Health.

[21] Gonçalves REM, Ponce JC, Leyton V (2018). Alcohol use by suicide victims in the city of Sao Paulo, Brazil, 2011–2015. J Forensic Leg Med.

[22] Schellenberg M, Hunt BL, Owattanapanich N, Jakob D, Lucas JR, Benjamin ER, Lewis M, Inaba K, Demetriades D. Hangings: Lessons learned from the coroner’s office. J Surg Res. 2021;264:158–62. 10.1016/j.jss.2021.02.021. 10.1016/j.jss.2021.02.021 33831602

[23] Stroebe W (2013). Firearm possession and violent death: a critical review. Aggress Violent Beh.

[24] Sarai SK, Abaid B, Lippmann S (2017). Guns and Suicide: are they related?. Prim Care Companion CNS Disord..

[25] Stack S, Rockett IRH (2018). Are suicide note writers representative of all suicides? Analysis of the national violent death reporting system. Suicide Life-Threat Behavior.

[26] Chávez-Hernández AM, Macías-García LF (2016). Understanding suicide in socially vulnerable contexts: psychological autopsy in a small town in Mexico. Suicide Life-Threat Behavior.

[27] Luke JN, Anderson IP, Gee GJ, Thorpe R, Rowley KG, Reilly RE, Thorpo A, Stewart PJ (2013). Suicide ideation and attempt in a community cohort of urban aboriginal youth: a cross-sectional study. Crisis: J Crisis Intervent Suicide Prev..

[28] Sheehan CM, Rogers RG, Boardman JD (2015). Post-mortem presence of drugs and method of violent suicide. J Drug Issues.

[29] Branas CC, Richmond TS, Ten Have TR, Wiebe DJ (2011). Acute alcohol consumption, alcohol outlets, and gun suicide. Subst Use Misuse.

[30] Anjos TG, Carvalho DSB, Machado AC, Carvalho MDSL, Lyrio AO, Souza ES, Gomes JA, Hintz AM, Cruz SS, Gomes-Filho IS, Figueiredo ACMG, Pereira MG (2021). Associated factors to abusive alcoholic beverage consumption in suicide victims. Drug Alcohol Depend.

[31] Larini L (1977). Toxicologia.

[32] Oquendo MA, Mann JJ (2000). The biology of impulsivity and suicidality. Psychiatr Clin North Am.

[33] Costanza A, Rothen S, Achab S, Thorens G, Baertschi M, Weber K, Canuto A, Richard-Lepouriel H, Perroud N, Zullino D (2021). Impulsivity and impulsivity-related endophenotypes in suicidal patients with substance use disorders: an exploratory study. Int J Ment Heal Addict.

[34] Hawton K (2000). Sex and suicide: gender differences in suicidal behaviour. Br J Psychiatry.

[35] Canetto SS, Sakinofsky I (1998). The gender paradox in suicide. Suicide Life-Threat Behavior.

[36] Raue PJ, Ghesquiere AR, Bruce ML (2014). Suicide risk in primary care: identification and management in older adults. Curr Psychiatry Rep.

[37] Lovisi GM, Santos SA, Legay L, Abelha L, Valencia E (2009). Epidemiological analysis of suicide in Brazil from 1980 to 2006. Braz J Psychiatry.

[38] Conner KR, Lathrop S, Caetano R, Wiegand T, Kaukeinen K, Nolte KB (2017). Presence of alcohol, cocaine, and other drugs in suicide and motor vehicle crash decedents ages 18 to 54. Alcoholism: Clin Exper Res..

[39] Zalsman G, Braun M, Arendt M, Grunebaum MF, Sher L, Burke AK, Brent DA, Chaudhury SR, Mann JJ, Oquendo MA (2006). A comparison of the medical lethality of suicide attempts in bipolar and major depressive disorders. Bipolar Disord.

[40] Brakoulias V, Starcevic V, Belloch A, Brown C, Ferrao YA, Fontenelle LF, Lochner C, Marazziti D, Matsunaga H, Miguel EC, Reddy YCJ, Rosario MC, Shavitt RG, Sundar AS, Stein DJ, Torres AR, Viswasam K (2017). Comorbidity, age of onset and suicidality in obsessive–compulsive disorder (OCD): an international collaboration. Compr Psychiatry.

[41] Goñi-Sarriés A, Blanco M, Azcárate L, Peinado R, López-Goñi JJ (2018). Are previous suicide attempts a risk factor for completed suicide?. Psicothema.

[42] Uribe IP, Blasco-Fontecilla H, García-Parés G, Batalla MG, Capdevila ML, Meca AC, Leon-Martinez V, Pèrez-Solà V, Vidal DJP (2013). Attempted and completed suicide: not what we expected?. J Affect Disord.

[43] VanSickle M, Werbel A, Perera K, Pak K, DeYoung K, Ghahramanlou-Holloway M (2016). Principal component analysis of the suicide opinion questionnaire in a U.S. military sample of Marine Corps Non-Commissioned Officers. Military Med.

[44] Stralea M, Krysinska K, Van Overmeirenc G, Andriessend K (2017). Geographic distribution of suicide and railway suicide in Belgium, 2008–2013: a principal component analysis. Int J Inj Contr Saf Promot.

[45] Costantini L, Pasquarella C, Odone A, Colucci ME, Costanza A, Serafini G, Aguglia A, Murri MB, Brakoulias V, Amore M, Ghaemi SN, Amerio A (2021). Screening for depression in primary care with Patient Health Questionnaire-9 (PHQ-9): a systematic review. J Affect Disord.

[46] Maertschi M, Costanza A, Canuto A, Weber K (2019). The dimensionality of suicidal ideation and its clinical implications. Int J Methods Psychiatr Res.

[47] Posner K, Brown GK, Stanley B, Brent DA, Yershova KV, Oquendo MA, Currier GW, Melvin GA, Greenhill L, Shen S, Mann JJ (2011). The Columbia-suicide severity rating scale: initial validity and internal consistency findings from three multisite studies with adolescents and adults. Am J Psychiatry.

