# Supplementary material for: Psychoactive substances use before suicide: detailed analysis of all cases that occurred in the Brazilian Federal District in a 10-years period
Source: BMC Psychiatry. 2022 Jul 14;22:467. doi: 10.1186/s12888-022-04082-z (PMC9284851; doi:10.1186/s12888-022-04082-z)
Supplement: Supplementary file 1 — Additional file 1: Diagram S1. Cases of suicide with toxicological examination were included in the study. Table S1. Parameters adopted for the variables studied. Brazilian Federal District, 2005 – 2014. Table S2. Results of principal component analysis showing factor loadings (correlation coefficients) and variance. Brazilian Federal District, 2005 – 2014. Figure S1. Three-dimensional PCA graph considering the first three principal components. Brazilian Federal District, 2005 – 2014. [file 12888_2022_4082_MOESM1_ESM.docx]

**SUPPLEMENTARY MATERIAL**

**TITLE:** Psychoactive substances use before suicide: detailed analysis of all cases that occurred in the Brazilian Federal District in a 10-years period

**DIAGRAM S1.** Cases of suicide with toxicological examination were included in the study.

* Psychoactive Substances

**Table S1.** Parameters adopted for the variables studied. Brazilian Federal District, 2005 – 2014

| **Variables** | **Parameters** |  | **Variables** | **Parameters** |
| --- | --- | --- | --- | --- |
| 1. Day of the month | From 1 |  | 11. Previous suicide attempt? | 0 – no |
|  | To 31 |  |  | 1 – yes |
| 2. Day of the week | 0 – weekday |  | 12. Recent behavior changed? | 0 – no |
|  | 1 – weekend |  |  | 1 – yes |
| 3. Month | From 1 – January |  | 13. Skin color | 0 – white people |
|  | To 12 – December |  |  | 1 – black/*pardo* |
| 4. Year | From 2015 |  | 14. Body Mass Index (BMI) | 0 – underweight |
|  | To 2014 |  |  | 1 – normal weight |
| 5. Period | 0 – day |  |  | 2 – overweight |
|  | 1 – night |  |  | 3 – obesity |
| 6. Age | 0 - 12-17 years old  1 – 18-29  2 – 30-59 |  | 15. Hanging | 0 – no |
|  | 3 – ≥60 |  |  | 1 – yes |
| 7. Sex | 0 – Female |  | 16. Firearm use | 0 – no |
|  | 1 – Male |  |  | 1 – yes |
| 8. Degree of education | 0 – illiterate |  | 17. Projection | 0 – no |
|  | 1 – elementary School |  |  | 1 – yes |
|  | 2 – high school |  | 18. Poison use | 0 – no |
|  | 3 – higher education |  |  | 1 – yes |
| 9. Marital status | 0 – not married |  | 19. Blood Alcohol Content (BAC) | From 0.02 |
|  | 1 – married |  |  | To 8.96 |
| 10. Suicide in your residence | 0 – no |  | 20. Drug use | 0 – no |
|  | 1 – yes |  |  | 1 – yes |

**Table S2.** Results of principal component analysis showing factor loadings (correlation coefficients) and variance. Brazilian Federal District, 2005 – 2014.

|  | Coefficients of PC1 | Coefficients of PC2 | Coefficients of PC3 | Coefficients of PC4 | Coefficients of PC5 |
| --- | --- | --- | --- | --- | --- |
| Day of the month | 0.10666 | 0.07381 | **-0.34262** | 0.07359 | **-0.47568** |
| Day of the week | -0.28072 | -0.10169 | 0.08138 | 0.29602 | 0.19667 |
| Month | 0.15248 | -0.09462 | -0.06067 | **0.30714** | -0.06015 |
| Year | 0.13308 | 0.24602 | -0.14355 | -0.12216 | **0.47216** |
| Period | -0.04197 | 0.04524 | **0.35882** | **0.32936** | **-0.46663** |
| Age | **0.3335** | **0.37386** | -0.20608 | 0.00833 | 0.00477 |
| Sex | **-0.38831** | 0.12438 | -0.00765 | 0.14945 | 0.2115 |
| Level of education | **0.36615** | **-0.33676** | 0.00048 | 0.03823 | 0.13981 |
| Marital status | 0.20274 | 0.25261 | 0.22332 | **0.37703** | 0.09128 |
| Suicide in own home | 0.05155 | 0.19284 | **-0.39194** | **0.37599** | -0.09189 |
| Previous suicide attempt? | 0.09861 | 0.26521 | **0.34433** | -0.18012 | -0.22014 |
| Recent behavior change? | 0.09873 | 0.27809 | 0.23418 | **-0.34877** | 0.10028 |
| Skin color | **-0.40057** | 0.13132 | 0.29211 | -0.08396 | -0.09474 |
| BMI | 0.28421 | -0.00874 | 0.29122 | 0.25197 | 0.29752 |
| Hanging | **-0.39915** | 0.28084 | -0.29099 | 0.12497 | 0.14406 |
| Alcohol use? | 0.05872 | **0.35708** | -0.12034 | -0.26613 | -0.1644 |
| Drug use? | -0.06164 | **-0.41435** | -0.18502 | -0.26602 | -0.07675 |
| % of variance | 9.47 | 8.40 | 7.60 | 7.56 | 6.90 |


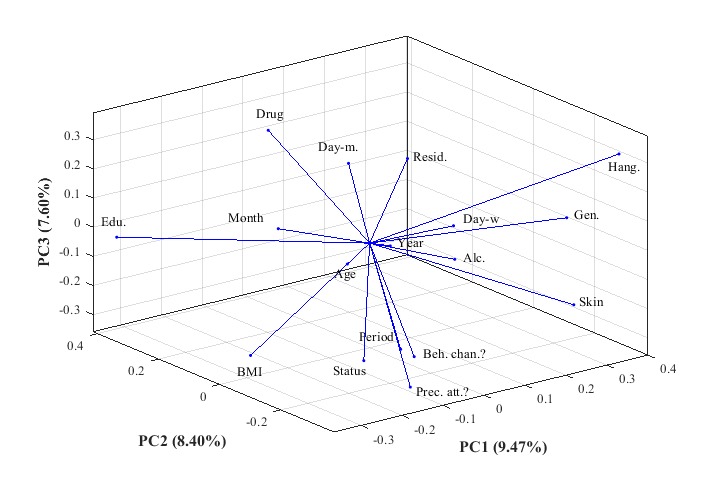


**Figure S1.** Three-dimensional PCA graph considering the first three principal components. Brazilian Federal District, 2005 – 2014
